# Research and Remedies

**Published:** 1913-03-29

**Authors:** 


### RESEARCH AND REMEDIES.

### Diabetic Perforating Ulcer.

The perforating ulcer of locomotor ataxia is familiar to every medical man and fifth-year student. A similar lesion occurs in diabetics, though it is not nearly so common, and is not mentioned in some of the text-books. In the *Johns Hopkins Hospital Bulletin* Sample and Gorham have collected seven such cases from 275 admissions for diabetes. After reviewing their cases and the possible explanations, they conclude that vascular change alone, pressure, and trophic neural disturbance may be contributory factors, but do not fully explain the lesion. They deem the loss of tissue vitality from hyperglycæmia to be an essential factor, and to this also they ascribe furuncles, carbuncles, and gangrene. Males are more commonly affected than females, and nearly every recorded case in the literature has been at forty years of age or upwards. The glycosuria is usually of a mild type, possibly connected with the elderly ages at which perforating ulcer alone appears. The diabetic ulcer is distinguished from that of locomotor ataxia by its much wider distribution; the former may occur on the sides or upper surface of the feet or on the hands, as well as on the soles, where alone the latter kind are commonly found. The knee-jerks are inconstant; all diabetics are liable to loss of this reflex, but it does not appear that there is any connection between this phenomenon and the occurrence of perforating ulcers.

### Tonsillotomy and Tonsillectomy.

After a long reign of favour with operators of all degrees of experience and dexterity, tonsillotomy has recently suffered severely from the competition of tonsillectomy, which many laryngologists have adopted within the last two or three years. There is no doubt whatever that tonsillectomy is a much more severe operation, and that it demands a greater amount of care both in its performance and in its after treatment; its risks, moreover, may develop into definite dangers unless the surgeon has self-reliance and foresight. On the other hand, it has certain advantages over tonsillotomy; and the determination of whether these advantages are as great in practice as they are theoretically and of the relative merits of the two operations has been undertaken lately in Mr. Harmer's clinic at St. Bartholomew's by Mr. Whale, whose detailed results are published in the *Lancet*. Statistics were taken very carefully and thoroughly of 110 patients who had been subjected to tonsillotomy and of 110 treated by tonsillectomy. The results are fully analysed, and they lead Mr. Whale to the following conclusions. The disadvantages of tonsillotomy are (1) the initiation of an infection of the tonsil or lymphatic glands, which is more likely if a free and deep removal has been performed than a moderate one; (2) recurrence of the trouble for which the operation has been done, except in the case of voice troubles. Tonsillectomy, in turn, is shown to be more liable to cause (1) harmful deformity, especially adhesion of the posterior faucial pillar to the pharyngeal wall, and (2) voice troubles, even apart from any definite deformity. Tonsillectomy is found to be the more dangerous of the two, but much the more likley to cure adenitis or tonsillitis. This research certainly justifies the statement that to those whose living depends in any way upon the use of the voice tonsillectomy should be urged with great caution.

### Researches on Pneumonia.

The firstfruits of Sir Almroth Wright's recent researches in South Africa have now appeared in some detail, and as might be expected they are extremely interesting. He has been investigating the action of a compound known as æthylhydrocupreinhydrochlorate, which Morgenroth had previously recommended as a specific for pneumonia. He concludes that this drug is very efficacious in the pneumococcic injections of mice; and that it renders the human blood fluids bactericidal. But it fails to exert any favourable influence on pneumonia in human beings. He offers two suggestions as to the possible cause of this failure, the most plausible of which is that the outpouring of lymph into the lung and its subsequent coagulation there affords a nidus for the bacteria in which the bacteriotropic fluids of the blood or tissues cannot get at them. We have here, Sir Almroth very justly writes, clearly a factor which would account for the success of Morgenroth's drug in mice, where the pneumococcic infection takes the form of a septicæmia; and for its failure in human pneumonia, where the drug would have only restricted access to the injecting microbes. But the pneumococcus does sometimes cause a septicæmia in man, and we hope Sir Almroth will see to it that the drug receives a fair trial in some such cases and report the results.

### Gastric Ulcer and Gastric Cancer.

In a paper which is a model of brief and scientific lucidity, Mr. Paterson explains in the *Lancet* his reasons for differing from the Mayos and several other authors concerning the prevalence of gastric ulcer in those who ultimately develop gastric cancer. Mayo, Graham, Wilson, M'Carty, and other authors in this country have been lately advocating the view that cancer of the stomach in a large percentage, even the majority, of cases originates in an old ulcer (or in the scar of one). Paterson exposes two fallacies in the chain of argument by which this conclusion is reached, and adduces further evidence which leads to an opposite belief—namely, that only some 1 to 2 per cent. of gastric ulcers ultimately become malignant. He holds that the subject requires further investigation, and that at present those who believe in the frequent causal relation of simple and malignant ulcer have not proved their case. His views are supported by some very telling arguments of an *a posteriori* kind, and will doubtless receive the consideration which his writings always deserve.

